# Physical and mechanical characterization of PLLA interference screws produced by two stage injection molding method

**DOI:** 10.1007/s40204-016-0056-4

**Published:** 2016-10-22

**Authors:** Ali Reza Sadeghi-Avalshahr, Mohammad Khorsand-Ghayeni, Samira Nokhasteh, Amir Mahdi Molavi, Mohammad Sadeghi-Avalshahr

**Affiliations:** 1 Department of Materials Research, Iranian Academic Center for Education, Culture and Research (ACECR), Mashhad Branch, Azadi Square, P.O. Box 91775-1376, Mashhad, Iran; 2 Materials Engineering Department, Tarbiat Modares University, Tehran, Iran; 3 Department of Mechanical Engineering, Tarbiat Modares University, Tehran, Iran

**Keywords:** Interference screw, Biodegradable, PLLA, ACL reconstruction

## Abstract

The purpose of this study was to produce and evaluate different mechanical, physical and in vitro cell culture characteristics of poly(L-lactic) acid (PLLA) interference screws. This work will focus on evaluating the effect of two important parameters on operation of these screws, first the tunnel diameter which is one of the most important parameters during the operation and second the thermal behavior, the main effective characteristic in production process. In this work, PLLA screws were produced by a two-stage injection molding machine. For mechanical assessment of the produced screws, Polyurethane rigid foam was used as cancellous bone and polypropylene rope as synthetic graft to simulate bone and ligament in real situation. Different tunnel diameters including 7–10 mm were evaluated for fixation strength. When the tunnel diameter was changed from 10 to 9 mm, the pull-out force has increased to about 12 %, which is probably due to the aforementioned frictional forces, however, by reducing the tunnel diameter to 8 and 7 mm, the pull-out force reduced to 16 and 50 % for 8 and 7 mm tunnel diameter, respectively. The minimum and maximum pull-out force was obtained 160.57 and 506.86 N for 7 and 9 mm tunnel diameters, respectively. For physicochemical assay, Fourier transform infrared spectroscopy (FTIR), degradation test and differential scanning calorimetry (DSC) were carried out. The crystallinity (Xc) of samples were decreased considerably from 64.3 % before injection to 32.95 % after injection with two different crystallographic forms α′ and α. probably due to the fast cooling rate at room temperature. In addition, MTT and cell attachment assays were utilized by MG63 osteoblast cell line, to evaluate the cytotoxicity of the produced screws. The results revealed no cytotoxicity effect.

## Introduction

Anterior cruciate ligament (ACL) reconstruction is the sixth most frequently performed procedure in orthopedics; however, many studies have been done in this field. Research topics cover different issues, mainly surgical technique factors such as tunnel position, graft choices, and fixation methods, as well as postoperative rehabilitation protocols. Due to many different biomechanical and clinical studies, interference screw fixation is the method of choice against all ACL graft fixation techniques (Prodromos et al. 2007; Dhillon et al. 2016).

Since Lambert (1983) introduced interference screw fixation of bone-patellar tendon-bone grafts, design, and performance of these screws have gradually improved. First generations of these screws were made by metallic biomaterials. To decrease the likelihood of graft laceration during insertion of the screw, designs with blunt threads have been developed. Cannulated screw designs made it possible using guide wires to minimize screw-tunnel divergence during insertion. But there were some complications after surgery such as pain requiring implant removal (Kurzweil et al. 1995), intra-articular migration (Sidhu and Wroble 1997), as well as difficulty in postoperative imaging. The advent of bioabsorbable interference screws has generated a great deal of interest and further research in graft fixation. These problems subsequently resulted to the advent of bioabsorbable interference screws in the early 1990s, which gained wide acceptance in graft fixation (Barber 1999). Some advantages of these bioabsorbable interference screws in comparison with metal screws include less interference with magnetic resonance images and so better postoperative imaging, less laceration of graft during insertion and easier revision surgery The disadvantages of these implants include screw breakage during insertion and soft tissue inflammatory reactions (Kaeding et al. 2005; Prodromos et al. 2007). Screw failure during insertion is related to some factors such as drive shape, length, and diameter as well as core diameter of the screw (Weiler et al. 2000). Poly(L-lactic) acid (PLLA), polyglycolic acid and their copolymers are the most common materials that are used by different manufacturers for producing of bioabsorbable interference screws.

Some different materials and methods have been investigated for graft fixation in ACL reconstruction. Barber et al. (Barber 2005) studied the clinical aspects of using poly-D, L-Lactide (PDLLA) interference screws; they concluded that these screws work well clinically, comparable to PLLA and metal interference screws. No data were provided for mechanical and physical characterization of these screws. Hunt and Callaghan (2008), carried out an in vitro animal study for the comparison of a composite (PLLA-HA) against PLLA screw. They concluded that the composite screw significantly increased new bone formation and decreased inflammatory reactions in comparison with the PLLA screw. Konan and Haddad (2009) studied 59 patients (average age was 34 years) for hamstring ACL reconstructions with polylactide carbonate (PLC) interference screws and concluded that the unpredictable screw degradation and the next body reaction to it can results in serious clinical outcomes.

In this work, we studied some different aspects which are important in the final performance of bioabsorbable interference screws including; tunnel diameter as one of the most important parameters in the surgical procedure as well as thermal behavior of produced screws as an important production process factor. Some PLLA interference screws were produced by a two-stage injection molding machine. The two-stage system has some advantages in comparison with reciprocating ones, such as shorter cycle time, more consistent melt quality, and more consistent shot size (Lim et al. 2008). Complete evaluations were carried out on produced samples, including mechanical and physical properties as well as in vitro cell cultures.

## Materials and methods

### Materials

The ester end-capped poly(L-lactic acid) (DG-L150, M_w_:130000, PDI:1.8) was provided from Jian Daigang Biomaterial (Republic of China). MTT (3-[4, 5-dimethylthiazol-2-yl]-2, 5 diphenyl tetrazolium bromide), Trypsin, and Ethylenediaminetetraacetic acid (EDTA) were purchased from Sigma-Aldrich (USA). Dimethyl sulfoxide (DMSO), Dulbecco’s Modified Eagle Medium (DMEM), Fetal bovine serum (FBS) were from Invitrogen (Germany). Simulated body fluid (SBF) and rigid polyurethane foams (PU) were purchased from Pardis research company (Iran).

### Fourier transform infrared spectroscopy (FTIR)

For assurance of nature and purity of polymer granules, a Shimadzu DR 8001 Model Fourier transform infrared spectrophotometer (FTIR) was used. The spectra were recorded between 400 and 4000 cm^−1^ with a spectral resolution of 2 cm^−1^ averaging 32 scans. Fourier transform infrared spectroscopy (FTIR) has been widely used for determining the elements in molecular structures, and for characterizing the synthesized compounds, from which the direct structural information and changes can be obtained during various chemical treatments.

### Screw fabrication

A special two-stage small injection molding machine was designed and fabricated for the production of 30 screws with 10 mm diameter (maximum injection pressure of 450 bar and maximum shot size of about 20 g). Like a traditional reciprocating screw machine, a two-stage injection machine utilizes a small screw to melt and convey material. However, unlike traditional machines, the screw is not responsible for injecting plastic into the mold. It feeds a second chamber, which is metered precisely, and then injected into the mold via a high-speed piston.

### Screw characterization

#### Differential scanning calorimetry (DSC)

To study the crystallization and melting behavior of PLLA samples, DSC measurement were carried out using a Perkin-Elmer (Pyris 1, USA), heating rate of 10 °C/min and scanned from 10 to 260 °C. DSC is a thermoanalytical method. In this technique, the difference for heat required for increasing the temperature of a sample and a reference is measured as a function of temperature. Both the sample and reference are maintained at the same temperature throughout the experiment. The reference sample should have a well-known heat capacity over the range of temperatures to be scanned (Wunderlich 2005).

#### Mechanical properties

##### Failure strength

**Fig. 1:**
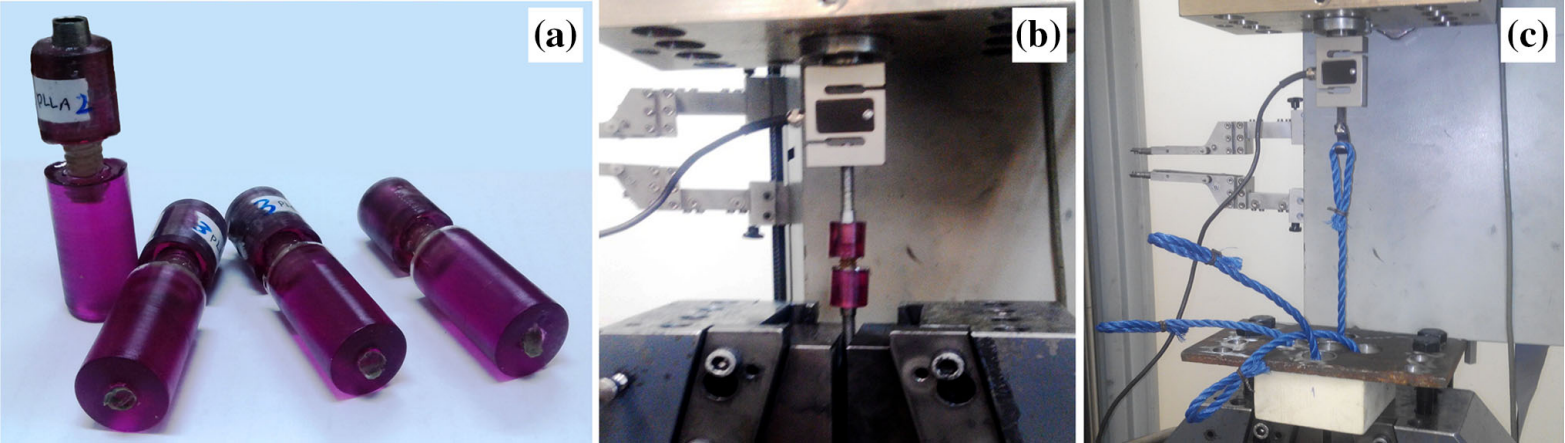
**a** Fixation of screws in the polyester resin for tensile testing, **b** fixation of screws in the tensile testing machine, and **c** determination method of fixation strength for screws

A universal testing machine (Zwick-Z250) with a 2KN load cell was used to evaluate the fracture strengths of the produced screws in tension mode. For a better simulation of practical conditions, it was tried to use the complete screws for these tests, so the screws were mounted in polyester resins together with some metal nut in both ends, according to Fig. 1a and b. Tests were performed at a strain rate of 30 mm/min until fracturing of screws. The tensile test was carried out after injection, and after immersion in FBS at 37 °C in an incubator for 2 and 4 weeks.

##### Insertion torque

Rigid polyurethane foam blocks with 0.2 g/cm^3^ density and dimensions of 10 × 10 × 5 cm were used as substitutes for cancellous bone (Bailey et al. 2006), according to the ASTM F1839. To determine the effect of tunnel diameter on initial fixation and required torque of insertion, four holes were created on each foam block with diameters of 7, 8, 9, and 10 mm. Polypropylene ropes with 8 mm diameter were used as graft substitutes. After placing the ropes in the holes, the insertion torque was measured using an analog torque meter (BRITOOL Company, Germany) during insertion of screws in the holes.

##### Fixation strength

The above-mentioned perforated foam blocks were used for the determination of fixation strength in this test. After placing the ropes into the holes and insertion of screws, the top side of the propylene ropes were looped and connected to a M8 hook, which was screwed to the load cell. The tensile test was carried out at the strain rate of 5 mm/min. A perforated metal sheet was used as a jig for these tests (Fig. 1c). The number of repetition was three for all of the above-mentioned tests.

#### In vitro degradation study

In vitro degradation studies were accomplished by immersing the screws in simulated body fluid (SBF), samples were immersed in SBF at 37 °C keeping a volume/mass ratio of 25/1 for 4 weeks. The degradation rate of the samples was evaluated by means of weight loss measurements (the number of repetition was three).

#### MTT assay

To determine the toxicity of samples and their effects on cell growth and proliferation, the process of extraction was performed according to ISO standard 10993-5 (ISO 10993 2009).

First, approximately 1 × 10^4^ cell were taken to each well of 96 wells cell culture plate and 100 µl culture medium was added to each of the wells. The plates were placed in an incubator for 24 h. After attachment of the cells and ensuring their health, the culture medium was removed completely and 90 µl of prepared extraction of samples, along with 10 µl of FBS were added to each cell.

A control sample was placed in the vicinity of the standard culture medium. The cell cultures were incubated for 24 h again. After 3 days, the whole environment is completely removed and 100 µl solution of MTT (0.5 mg ml^−1^) was added to the wells. The culture plates were incubated for 4 h. After this time, MTT reaction medium was removed and 100 µl isopropanol (Sigma, USA) was added to each well to dissolve the resulted dark blue sediments of MTT reactions. For better dissolution of the precipitated crystals, the plates were placed on the shaker for 15 min. Finally, using ELISA (STAT FAX 2100, USA) in the wavelength of 545 nm, the absorbance was measured. Higher optical densities (OD) represent more viability of the cells and so the toxicity percent can be obtained from the following formula:

1 $$ {\text{Toxicity}}(\% ) = \left(1 - \frac{{{\text{OD}}_{\text{S}} }}{{{\text{OD}}_{\text{C}} }}\right) \times 100 $$

where OD_S_ is mean OD of sample and OD_c_ is mean OD of control.

These tests were done in MG63 cell line in Pasteur Institute of Iran and extracted three times by 1, 7 and 14 days. The Samples were sterilized prior to testing in an autoclave at 121 °C for 15 min (the number of repetition was three).

#### Cell attachment

Circular discs of samples were prepared, sterilized, and placed in 6 well plates in triplicates. The MG63 osteoblast cells with a density of 10^4^ cells/well were seeded onto the samples as explained already for MTT assay and cultured in the incubator at 37 °C and 5 % CO_2_. After 24 h, unattached cells were washed three times with PBS buffer and attached cells were fixed in 4 % glutaraldehyde at 4 °C for 24 h. Then, the samples were rinsed in double distilled water and dehydrated with ethanol for 15 min. After drying, to determine the adhesion of cells scanning electron microscopy (SEM) was used.

### Statistical analysis

All data were expressed as mean ± S.D. Data are subject to one-way analysis of variance (ANOVA), a value of *p* < 0.05 considered to be statistically significant.

## Results

### Mechanical properties

#### Failure strength

**Fig. 2:**
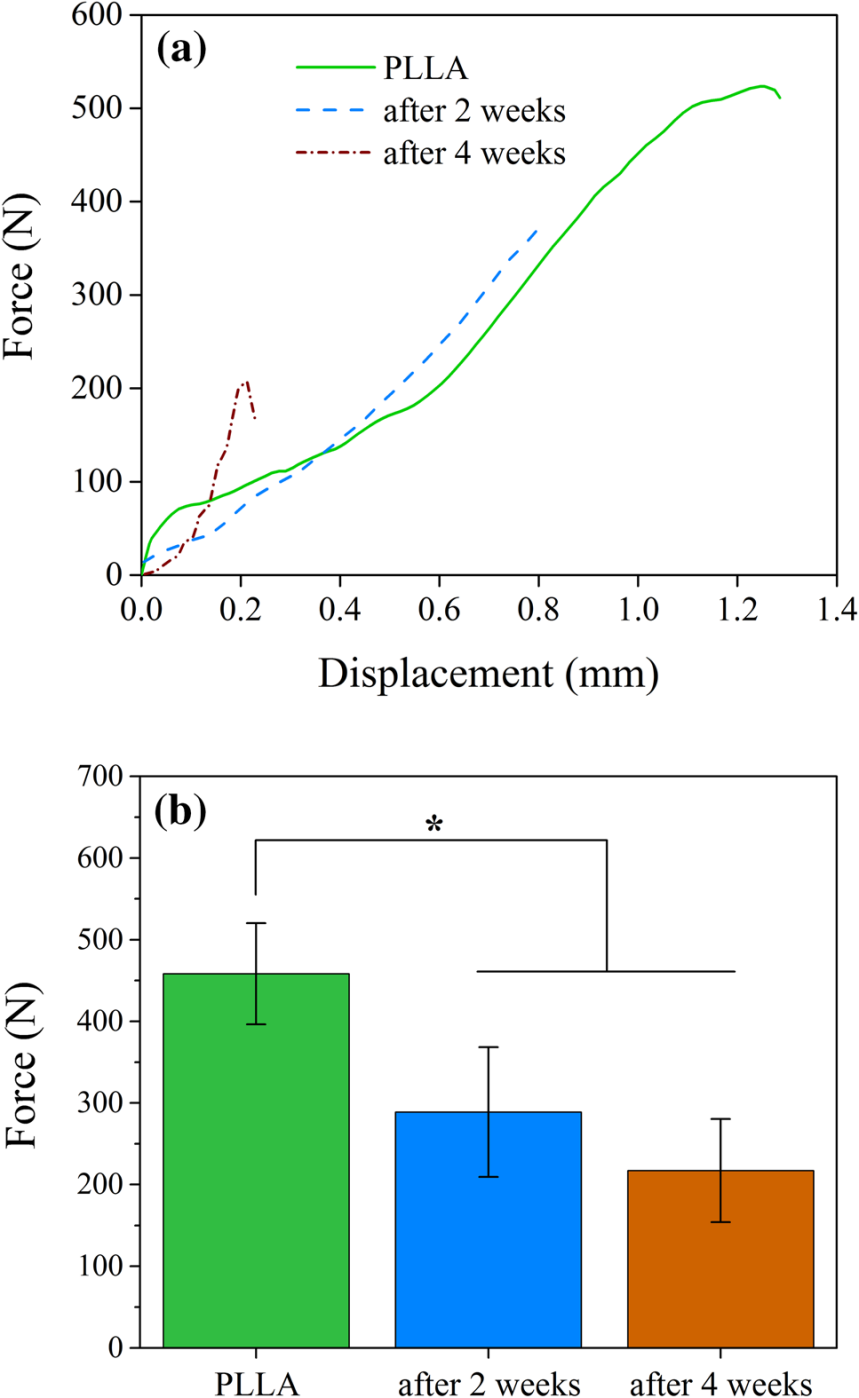
The results of mechanical strength before and after degradation times. **a** Force–displacement, **b** the maximum force. *p < 0.05 (*n* = 3)

The results of fracture strength of screws in this study, before and after immersion in SBF for 2 and 4 weeks are shown in Fig. 2.

It can be seen that the mechanical behavior has been changed from a tough material to a brittle one (Fig. 2a) and the maximum force (Fig. 2b) decreased with increasing of degradation time which can be due to the destruction of polymer structure in SBF.

#### Insertion torque

**Fig. 3:**
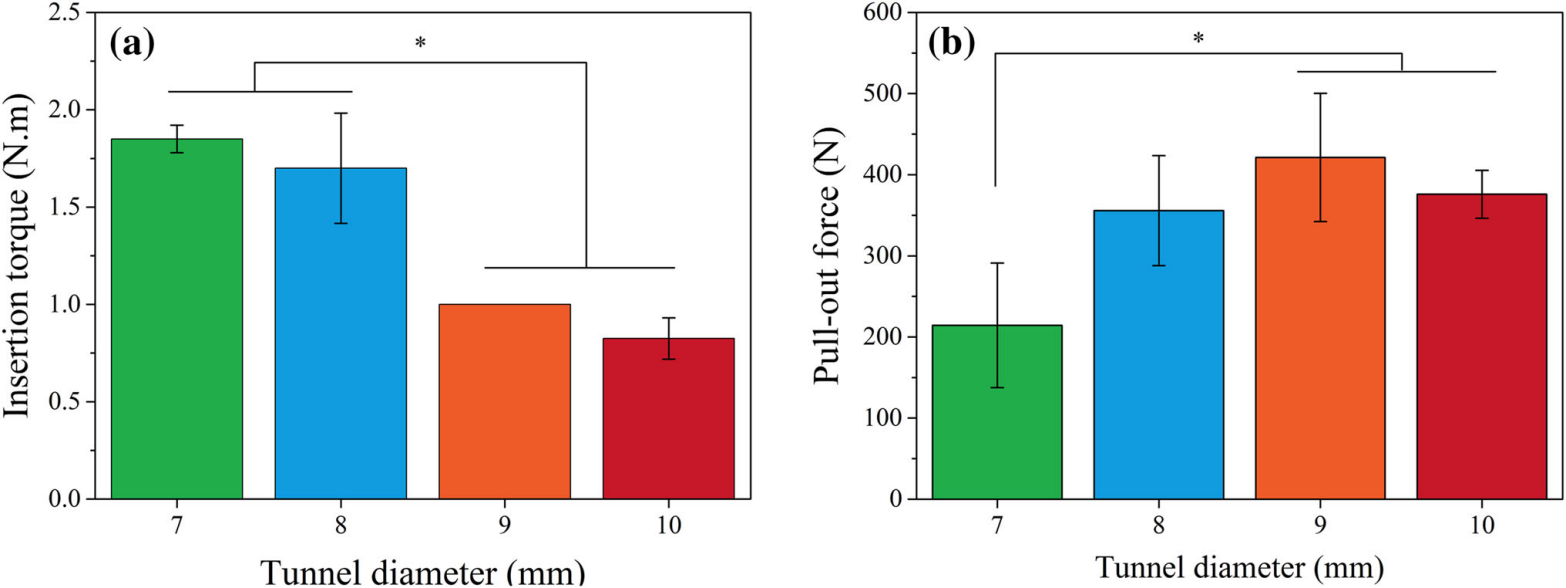
**a** The results of insertion torque, and **b** the results of pull-out strength. *p < 0.05 (*n* = 3)

Based on the review of the literature, the gap size between the bone block and bone tunnel is probably the most important factor, which affects the initial fixation properties of interference screw for fixation of bone-tendon-bone grafts (Prodromos et al. 2007). The result of this test can be seen in Fig. 3a and Table 1. As expected, by decreasing the diameter of the hole the amount of insertion torque increased.

**Table 1:** The results of fixation strengths and insertion torques for PLLA screws in different hole sizes

Hole diameter (mm)	Fixation strength (N)			Insertion torque (N m)		
7	180.24	160.57	302.39	1.8	1.8	1.9
8	334.28	301.29	431.66	1.6	1.5	1.9
9	405.67	351.21	506.86	1.1	1.0	1.0
10	387.95	342.34	397.59	0.7	0.8	0.9

*p* < 0.05, *n* = 3

#### Pull-out strength

The results are shown in Fig. 3b and Table 1. It has been estimated that the ACL is loaded from 27 N to 454 N during activities of daily living (Noyes et al. 1984). As it can be seen, the maximum pull-out force can be obtained for tunnels with 9 mm diameter and has been decreased to minimum amount for 7 mm diameter.

### DSC measurements

**Fig. 4:**
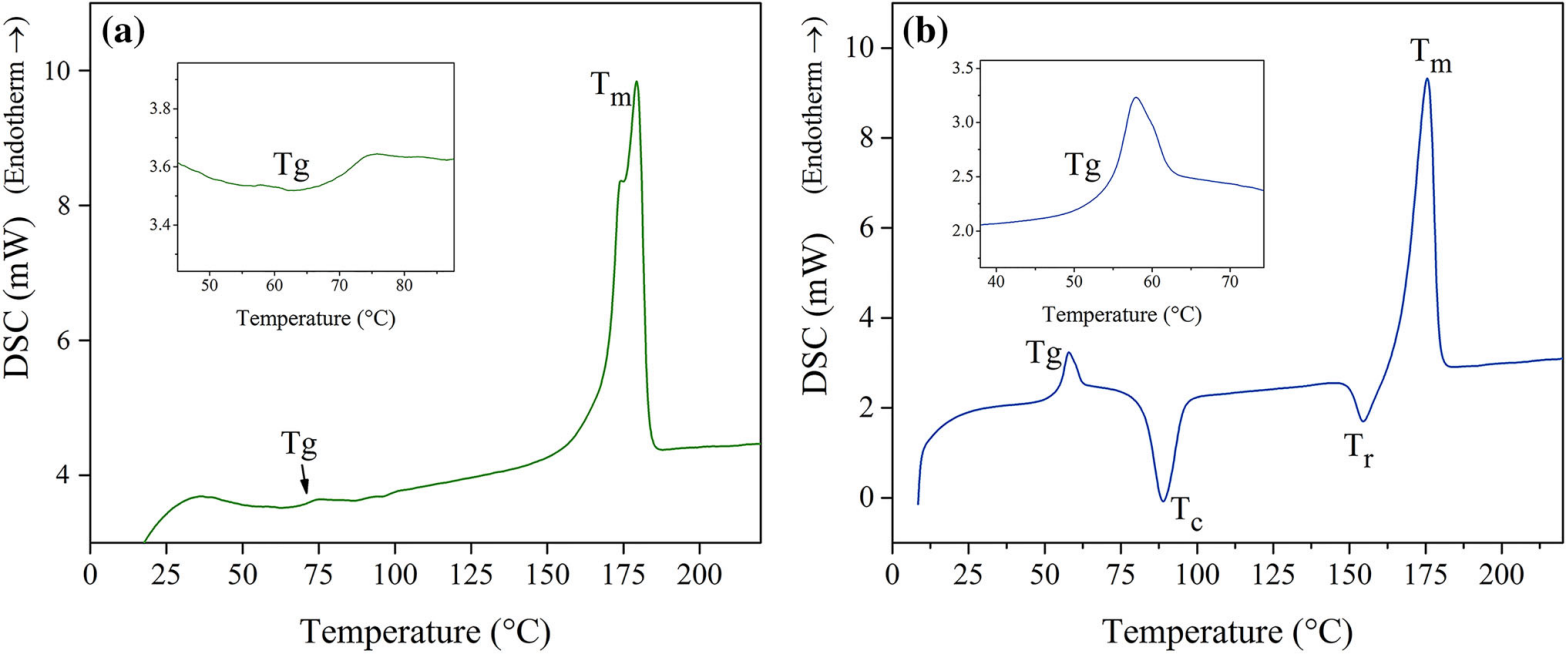
DSC curves for PLLA **a** before injection, **b** after injection

The DSC results can be seen in Fig. 4 and Table 2. *T*_g_ is the glass transition temperature, *T*_C_ is the temperature of cold crystallization peak, *T*_r_ is the temperature of recrystallization peak and *T*_m_ is the melting temperature. Δ*H*_C_ is the enthalpy of cold crystallization, and Δ*H*_m_ is the enthalpy of fusion, both of them normalized to unit mass of PLLA matrix. Also, the Δ*H*_m_^∞^ is the enthalpy of fusion of 100 % crystalline PLLA (93 J g^−1^) (Pantani and Sorrentino 2013). The degree of crystallinity of the samples, *X*_C_ % in Table 2 was obtained from Eq. 1 as follow (Rasselet et al. 2014):

**Table 2:** DSC results for PLLA before and after injection

	*T*_g_ (°C)	*T*_cc_ (°C)	−Δ*H*_cc_ (J g^−1^)	*T*_mr_ (°C)	−Δ*H*_mr_ (J g^−1^)	*T*_m_ (°C)	Δ*H*_m_ (J g^−1^)	*X*_c_ (%)
PLLA-before	66.35	–	–	–	–	168.65	59.79	64.30
PLLA-after	49.39	82.27	21.69	149.39	5.42	166.60	52.33	32.95

*p* < 0.05, *n* = 3

2 $$ X_{C} (\% ) = \frac{{\Delta H_{\text{m}} - \Delta H_{\text{CC}} }}{{\Delta H_{\text{m}}^{\infty } }} \times 100 $$

### Fourier transform infrared spectroscopy (FTIR)

**Fig. 5:**
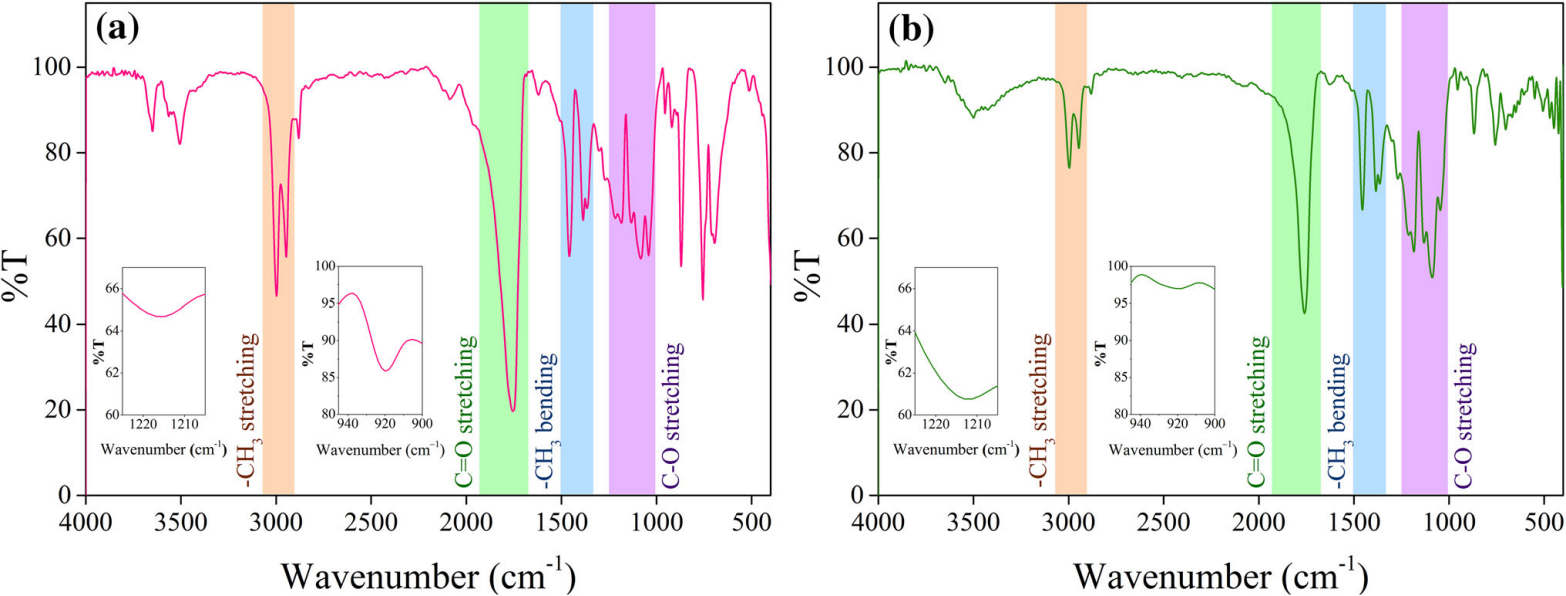
The results of FTIR spectroscopy for PLLA **a** before injection, **b** after injection

The result of FTIR is shown in Fig. 5. According to the Fig. 5a, a characteristic strong peak at about 1755 cm^−1^ is related to the stretching vibration of the carbonyl group. Two middle Peaks at 2997 and 2947 cm^−1^ (–CH_3_ symmetric and asymmetric stretching, respectively), two middle peaks at 1458 and 1362 cm^−1^ (–CH_3_ asymmetric and symmetric bending vibration, respectively), and a doublet peak at 1215/1184 cm^−1^ and a triplet peak 1132/1083/1041 cm^−1^ (C–O stretching vibration) indicates the nature of ester end-capped PLLA (Chieng et al. 2013).

The absorption bands at 920 cm^−1^ (owing to flexural C–H bond vibration) and 1213 cm^−1^ (owing to alkyl-ketone chain vibration) are representative of the crystalline structure of PLA (Jamshidian et al. 2010; Carrasco et al. 2010; Gorrasi and Pantani 2013).

### Degradation

**Fig. 6:**
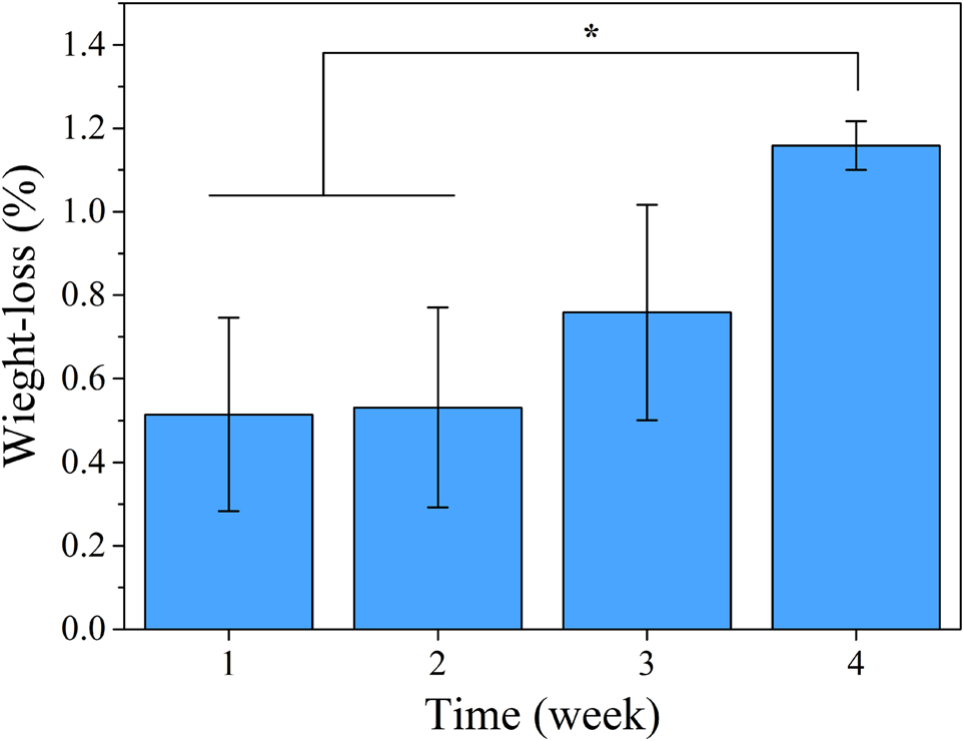
The weight loss of PLLA screw. *p < 0.05 (*n* = 3)

The results of the degradation test for this work are shown in Fig. 6. Degradation of PLLA occurs very slowly (Shasteen and Choy 2011) and it can be seen in Fig. 6 that the weight-loss of PLLA is only 1.2 % after 4 week. As it can be seen, first, the rate of destruction of PLLA samples is low and during the time, it has been increased.

### MTT assay and cell attachment

**Fig. 7:**
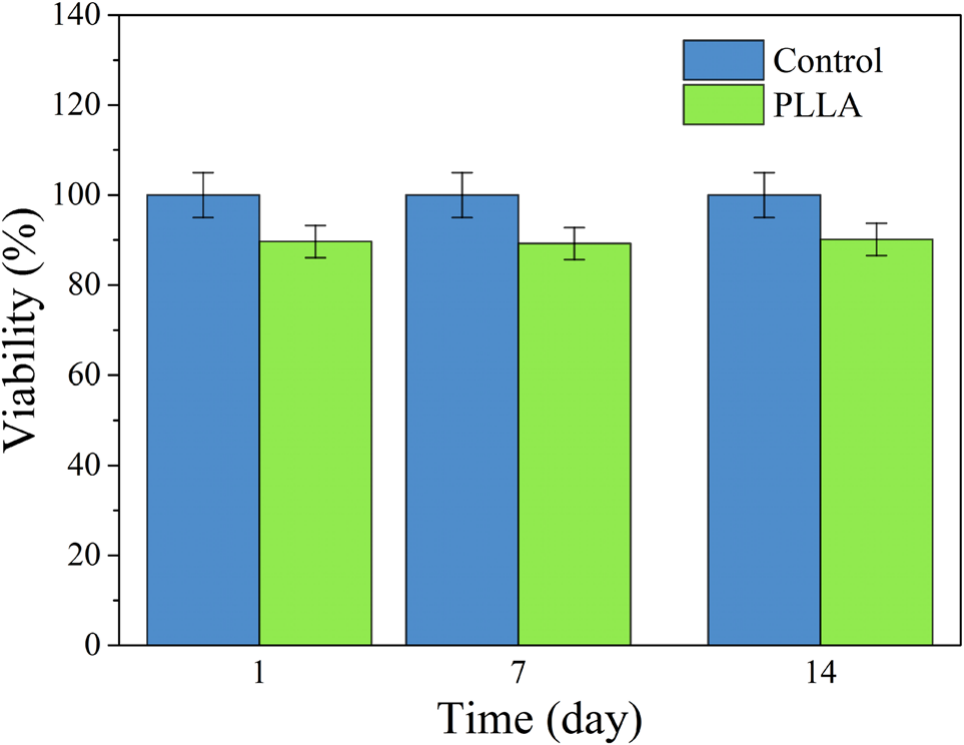
The results of MG63 cell viability in MTT assay. *p* < 0.05 (*n* = 3)

**Fig. 8:**
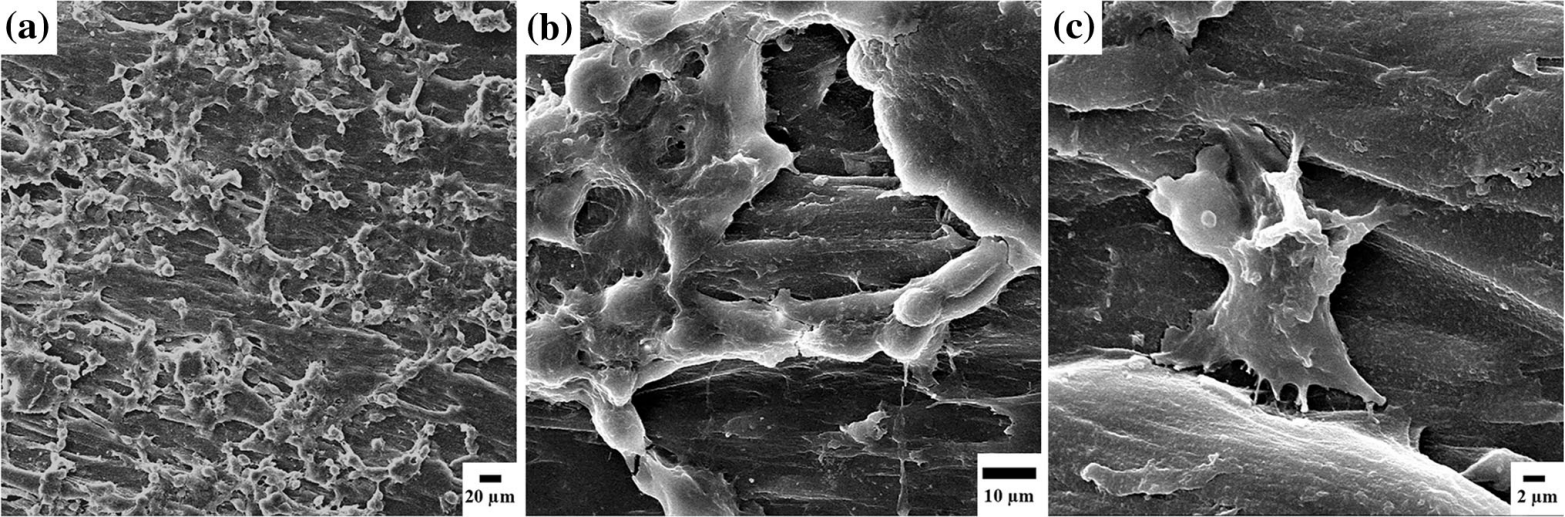
Attachment of MG63 osteoblast cells on the surface of PLLA screws

The results of cell viability are shown in Fig. 7. As it can be seen, the cell viability is about 85 % and is almost constant during the time and there is not any special evidence of toxicity at different stages of testing. In addition, The SEM images of MG63 cell attachments can be seen in Fig. 8, which represents good spreading of cells on the surface of PLLA samples.

## Discussion

### Mechanical properties

Using different research models and biomechanical testing methods make it hard to compare the results of various works with each other. Different biomechanical studies have compared the initial fixation strength of metal and bioabsorbable interference screws in animal and human cadaveric models and concluded that there are no significant differences in their mechanical performance (Kousa et al. 2001).

During the ACL reconstruction surgery, usually smaller tunnel diameter than the screw diameter is drilled into the bone to provide better fixation strength. In fact, the frictional force between the peripheral surface of the screw thread and the inner surface of tunnel plays an important role for this purpose. In this study, for the 10 mm diameter produced screw, different tunnel diameters including 7, 8, 9 and 10 mm were selected and drilled into the PU foams to evaluate the best condition for fixation strength. As it can be seen in Fig. 3b, it seems that there is an optimum condition for pull-out force. When the tunnel diameter was changed from 10 to 9 mm, the pull-out force has increased to about 12 %, which is probably due to the aforementioned frictional forces, but by mostly reducing the tunnel diameter to 8 and 7 mm, the pull-out force reduced to 16 and 50 % for 8 and 7 mm tunnel diameter, respectively. It seems that low density and low mechanical properties of PU test blocks have resulted in dilation of holes during insertion of screws in smaller holes and so unexpectedly have reduced the pull-out forces. In addition, the screw dislocation in the test blocks can affect the value of pull-out force during insertion. On the other hand, as it can be seen in Fig. 3a, with increasing the tunnel diameter the insertion torque has decreased. It seems that any parameter that decreases the frictional force in screw insertion will decrease the insertion torque (Battula et al. 2008).

### Evaluating the injection process by DSC

As it can be seen in Table 2, the crystallinity of injection molded screws has decreased considerably after injection compared to crystallinity before injection which could be due to the rather fast cooling rate at room temperature after injection. There are different structures for PLLA due to different conditions of crystallization such as temperature range and heating rate. Crystallization at temperatures more than about 120 °C results in a formation of α-crystals with two antiparallel aligned helical chain segments packed in an orthorhombic unit cell. At temperatures lower than about 120 °C, formation of these crystals is replaced by formation of pseudohexagonal α′-crystals which is considered as a conformationally disordered α-crystal with slightly increased lattice spacings, and turns into stable α-form upon heating at rates slower than 30 KS^−1^ (Pan et al. 2008; Zhang et al. 2008; Saeidlou et al. 2012; Androsch et al. 2014).

Considering the DSC curves in Fig. 4b, it seems that due to the low cooling rate in the mold and rather a fast cooling rate after that at room temperature, there is a mixture of α′ disordered and α ordered crystals in the structure. The exothermal peak just before the melting point in Fig. 3b could be due to the disorder-to-order phase transition of α′ to α. Meanwhile, a shoulder melting peak before the main melting peak in Fig. 4a could be due to the melting of these two different crystallographic forms α′ and α (Zhang et al. 2008).

### FTIR spectra

**Fig. 9:**
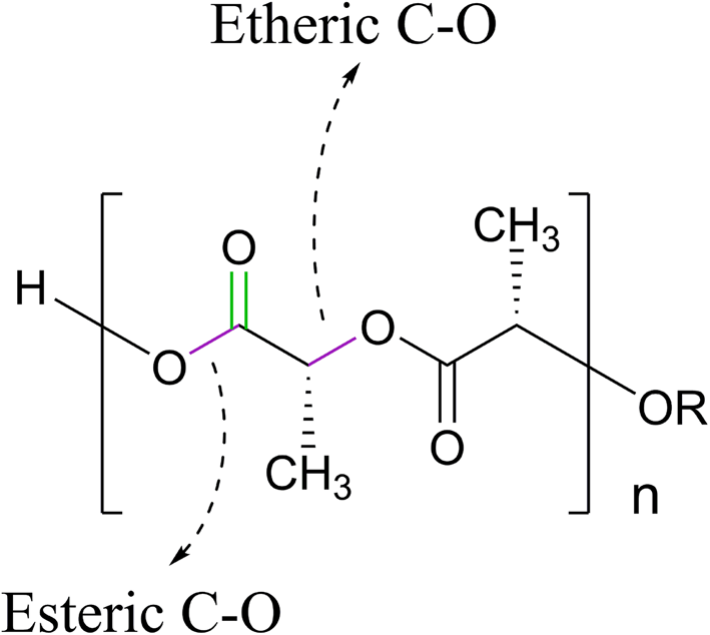
Schematic of chemical bonds in the PLLA backbones

As it can be seen in Fig. 9, the etheric and esteric C–O bonds of PLLA have stretching vibrations that occur in the same region (1000–1300 cm^−1^). The overlapping of these peaks can lead to peak broadening and branching. The C = O stretching vibration occurs at 1755 cm^−1^ and –CH_3_ bending and stretching observed in the range of 1350–1500 cm^−1^ and 2900–3000 cm^−1^, respectively. These results indicate the purity of ester end-capped PLLA.

Also, it can be seen from Fig. 5a and b that, the intensity of absorption bands after the injection has decreased for most peaks, which is inconsistent with the qualitative variation of crystallinity and DSC results. It can be inferred that the PLLA sample after the injection has less crystallinity due to the faster cooling rate during the injection process. The polymer chains did not allow rearrangement and forming of crystalline structure. Therefore, the intensity of two absorption band in the 920 and 1213 cm^−1^ regions have decreased.

### Degradation

Degradation of Polyester chains occurs due to the nature of hydrolysis reaction of ester bonds in the polymer backbone. When a water molecule attacks to a polyester chain, a reversible reaction occurs and polyester converts to initial hydroxyl and carboxyl groups (Sultana 2011). As a result, the average length of polymer chains becomes shorter and molecular weight of polymer decreases (Gleadall 2015). However, usually the longer remained chains in the polymer matrix prevent the outflow of short oligomers (Armentano et al. 2010). Remaining oligomers will decrease the pH value of these areas and cause a process that called autocatalytic degradation or acid catalytic degradation (Díaz-Gómez et al. 2015). Therefore, the weight loss of PLLA screw increases drastically by passing the time. Finally, the resultant monomers enter the Krebs cycle and excreted in the form of carbon dioxide and water (Sultana 2011; Gleadall 2015). As it can be seen in Fig. 2a, since the area under the curve used as an indicator of polymer toughness (Menard 2008), it can be concluded that with increasing the degradation time, the toughness decreases as a result of decreasing the polymer molecular weight during the degradation. This is in consistency with reducing of mechanical strength after immersion in SBF. As it can be seen in Fig. 2b, the fracture force has decreased to about 37 and 52 %, respectively, after 2 and 4 weeks immersion in SBF.

## Conclusions

Considering some important technical parameters in a surgical procedure as well as the effects of the production process on final properties and performance of bioabsorbable interference screws were studied in this work. Evaluating the thermal behavior of the produced screws revealed that the crystallinity of structure was decreased considerably after injection molding of screws due to the fast cooling rate at room temperature and it seems that there was two different crystallographic α′ and α form in the structure. In addition, it was concluded that the maximum pull-out strength of the produced screws could be achieved for 9 mm tunnel diameter.
